# Kinship of conditionally immortalized cells derived from fetal bone to human bone-derived mesenchymal stroma cells

**DOI:** 10.1038/s41598-021-90161-2

**Published:** 2021-05-25

**Authors:** S. Marozin, B. Simon-Nobbe, S. Irausek, L. W. K. Chung, G. Lepperdinger

**Affiliations:** 1 Department of Biosciences, University of Salzburg, Hellbrunnerstr. 34, 5020 Salzburg, Austria; 2 Cedars-Sinai Medical Center, Dept. of Medicine, 8700 Beverly Blvd b106, Los Angeles, CA 90048 USA

**Keywords:** Cell biology, Stem cells

## Abstract

The human fetal osteoblast cell line (hFOB 1.19) has been proposed as an accessible experimental model for study of osteoblast biology relating to drug development and biomaterial engineering. For their multilineage differentiation potential, hFOB has been compared to human mesenchymal progenitor cells and used to investigate bone-metabolism in vitro. Hereby, we studied whether and to what extent the conditionally immortalized cell line hFOB 1.19 can serve as a surrogate model for bone-marrow derived mesenchymal stromal cells (bmMSC). hFOB indeed exhibit specific characteristics reminiscent of bmMSC, as colony formation, migration capacity and the propensity to grow as multicellular aggregates. After prolonged culture, in contrast to the expected effect of immortalization, hFOB acquired a delayed growth rate. In close resemblance to bmMSC at increasing passages, also hFOB showed morphological abnormalities, enlargement and finally reduced proliferation rates together with enhanced expression of the cell cycle inhibitors p21 and p16. hFOB not only have the ability to undergo multilineage differentiation but portray several important aspects of human bone marrow mesenchymal stromal cells. Superior to primary MSC and osteoblasts, hFOB enabled the generation of continuous cell lines. These provide an advanced basis for investigating age-related dysfunctions of MSCs in an in vitro 3D-stem cell microenvironment.

## Introduction

Stromal cells have been extensively studied, also in regard to the corresponding stem cell type, which are most often called mesenchymal stem cells (MSC) as introduced by Arnold Caplan^1^. According to the “The International Society for Cell and Gene Therapy”, the former represents a cell population with secretory, immunomodulatory and homing properties, while the latter is considered a tissue-borne stem cell population with the ability to self-renew and generate progenitor cells prone to differentiate into various mesenchymal cell types^2^. Many studies have referred to the potential capability of this stem cell type in remodeling and regeneration of musculoskeletal tissues. For experimental analyses, primary MSC can be isolated from almost any mesenchymal tissue. However after explantation, and later on, during culture amplification, they undergo changes, which by and large resemble commonly known cellular aging phenotypes in conjunction with changes that are most likely due to donor-age and tissue of origin^3^. These undesirable and equally unpredictable deviations hampered up till now basic research in MSC biology.

Provided that, MSC biology may greatly benefit from the availability of a well-established in vitro MSC surrogate model. Several cell culture models have been proposed for studying bone formation in vitro. Immortalized and tumor cell lines have the advantage to provide an unlimited source of material and offer experimental reliability and reproducibility^4^. Some of the available cell models are also of human origin. Often they are derived from osteosarcomas, such as the cell line MG63 or SAOS-2^5^. The cancerous origin of these cells constrains comparative studies for a better understanding of bone formation and osseous degeneration under physiological conditions. Despite many advantages, several studies concluded that such cell lines are inadequately representing the dynamic properties of normal cells in vivo, primarily due to their malignant nature and altered cellular physiology^6^.

In order to circumvent the Hayflick limit, the catalytic component of human telomerase (hTERT) has been expressed in primary MSC. As a consequence thereof, life-span of MSC can be significantly increased, notably without limiting their inherent multi-lineage differentiation potential^7,8^. Besides retaining the parental cell properties, hTERT expressing MSC may escape in vitro senescence^9^. However, some minor indications of potential malignant transformations and chromosomal instability limited the use of various MSC lines that had been immortalized by means of constitutively expressing hTERT^10–12^.

As a matter of fact, MSC subjected to replicative or biological senescence show functional alterations, such as impaired differentiation potential and a decline of migratory ability^3,13^. Upon aging, bone-marrow MSC display a biased differentiation into adipocytes at the cost of osteoblasts. Divergent lineage fate decision with respect to adipogenesis over osteogenesis is believed to contribute to aging of mesenchymal tissue but the underlining molecular mechanisms are still unclear^14,15^.

In order to circumnavigate the aforementioned obstacles, we here investigated an osteoblastic non-tumorous cell line (hFOB1.19), which can be continuously expanded, thereby retaining both osteoblastic and adipogenic differentiation potentials. Our observations and analyses showed a high resemblance to MSC derived from adult donors, thus prompting that hFOB1.19 can be considered an appropriate cell model, which is amenable to solve still unanswered questions in MSC biology^16,17^.

## Results

### General properties of normal and transgenic human fetal osteoblast cells, hFOB1.19

**Figure 1:**
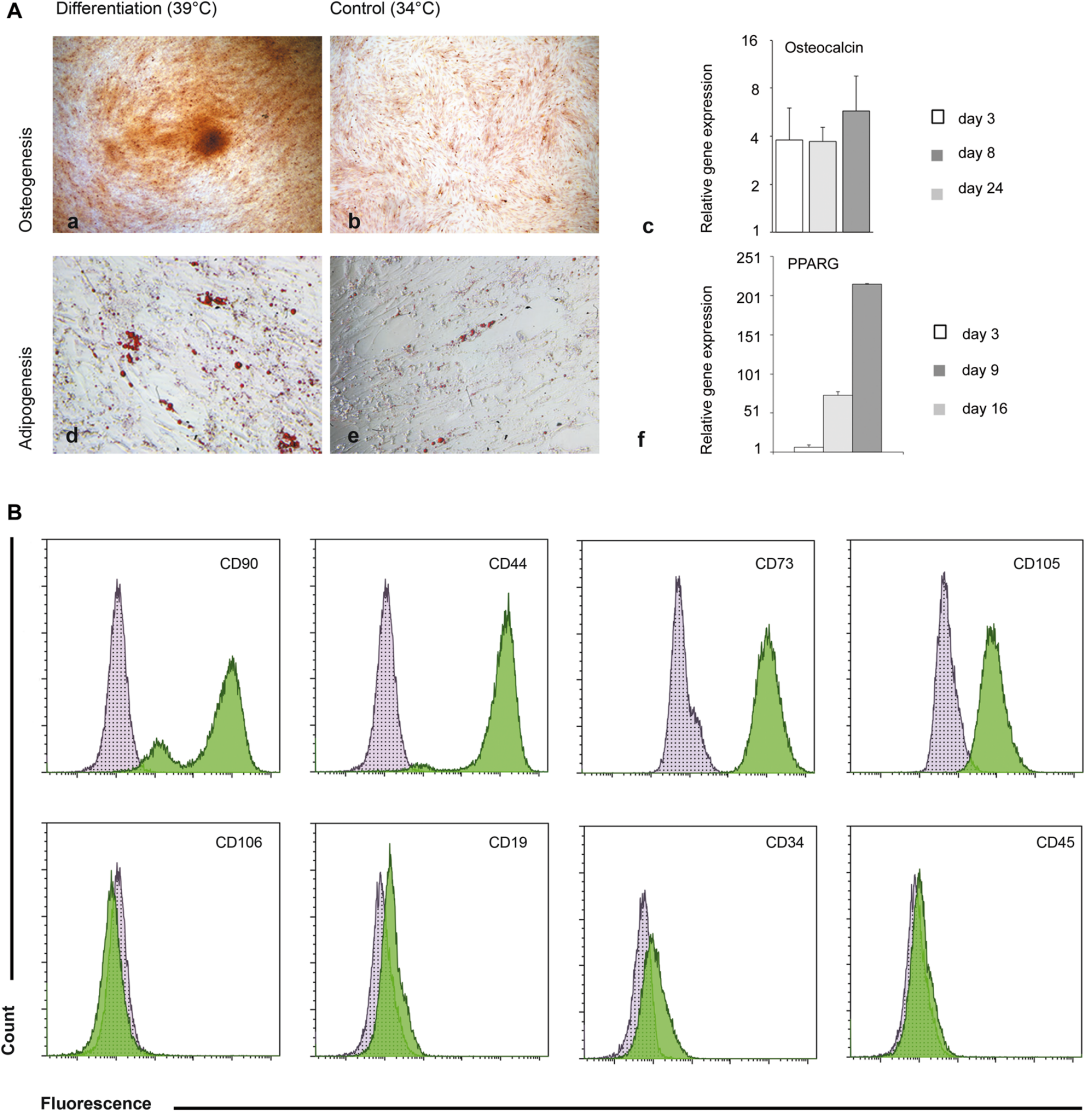
Characterization of hFOB1.19 cells. (**A**) Differentiation capacity towards the osteogenic lineage was tested after cell seeding at a density of 1 × 10^5^ cells/cm^2^ in DMEM:F12 medium at 34 °C and 5% CO_2_/20% O_2_. (a) The following day osteogenic differentiation was induced by shifting the temperature to 39 °C. After 24 days, cells were stained with Alizarin Red S. (b) As a control, cells were cultivated at 34 °C for 24 days and also stained with Alizarin Red. (c) Corresponding qPCR of the osteogenic marker (Osteocalcin) was normalized to YWHAZ (Tyrosine 3-monooxygenase) gene. (d) In order to examine adipogenic differentiation capacity, cells were seeded at a density of 1.5 × 10^5^ cells/cm^2^ in DMEM:F12 medium at 34 °C and 5% CO_2_/20% O_2_. Adipogenic differentiation was induced by shifting the temperature to 39 °C and supplementing the medium with the differentiation mix (0.5 mM isobutyl-methylxanthine, 1 µM dexamethasone, 10 µM insulin, 60 µM indomethacin) for 16 days. (e) Adipogenic control cells were incubated in DMEM:F12 medium at 34 °C and 5% CO_2_/20% O_2_ for 16 days. (f) Expression levels of the adipogenic marker (PPARg) was assessed by qPCR. For normalization YWHAZ (Tyrosine 3-monooxygenase) was used as a reference gene. (**B**) Cultures of hFOBs at 34 °C, 20% O_2_ were subjected to surface antigen analysis by flow cytometry. Flow cytometry was performed using Cytoflex S and the analysis performed with Kaluza software (Beckman-Coulter). Surface antigen in green and isotype control in grey.

Albeit predetermined to the osteogenic lineage, hFOB1.19 cells have been reported to also undergo adipogenic differentiation^17^. Exposure of hFOB cultures to 39 °C resulted in spontaneous osteogenic differentiation as mineralized nodules were formed, which readily incorporated Alizarin Red and transcription of the osteogenic-specific markers became activated (Fig. 1A; Fig. S1). Adipogenesis was successfully achieved during 16 days at 39 °C in the presence of adipogenic medium, as confirmed by the accumulation of intracellular lipid droplets, which were detected by Oil Red staining. This finding was also supported by transcriptional upregulation of the adipogenic factors PPARγ. hFOB also share multiple surface markers with bone-marrow stem cells (Fig. 1B). Naive hFOB were positive for CD90, CD44, CD73 and CD105, while negative for CD106, CD19, CD34 and CD45. Additionally, hFOB cells were positive for CD146 (Fig. S2).

**Figure 2:**
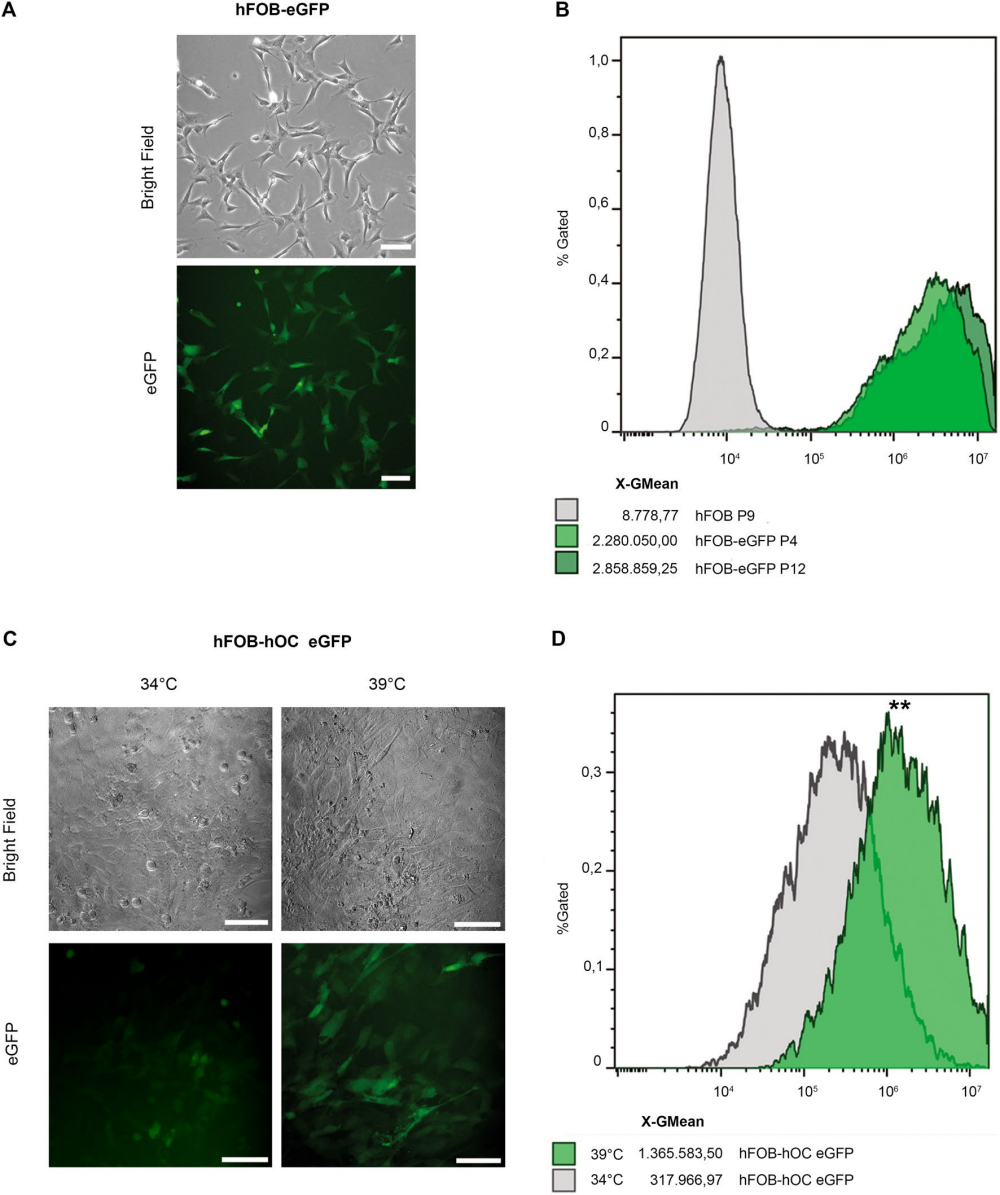
Transgenic hFOB reporter cell lines. (**A**) hFOB cells were transduced with a recombinant lentivirus vector bearing the reporter construct UbC-eGFP resulting in the cell line hFOB-eGFP. Constitutively active expression of eGFP was visualized by fluorescence microscopy. Bars indicate 100 µm. (**B**) Stable eGFP expression over cell passaging was assessed by flow-cytometry comparing wild-type hFOB (in grey) with hFOB-eGFP (in green) at low (P4) and high cell passage (P12). Data acquisition and analysis were performed by means of Cytoflex S, software CytExpert and Kaluza. (**C**) An inducible cell line expressing eGFP under the control of the full length promoter for the human osteocalcin (hFOB-hOC eGFP) was transduced and differentiation was induced by incubation at 39 °C for 3 days. Images of cells incubated at 34 °C (undifferentiated control) versus induced cultures at 39 °C were taken to monitor eGFP expression. (**D**) Representative histograms of flow-cytometric analysis of reporter gene expression in hFOB-hOC eGFP activated at 39 °C (green) for 3 days. Uninduced cells cultured at 34 °C (in grey) displayed a basal expression of eGFP. Geometric mean of fluorescence intensity is indicated. Statistical significance of reporter activity at 39 °C of three independent experiments was calculated using an unpaired Student *t* test.

Primary mesenchymal stromal cells, which exhibit osteoblastic potential display a finite capacity to replicate in vitro, in particular when explanted from donors of advanced age. Often they can only be cultured for a limited time before they undergo senescence^18,19^. For this reason, generation of stable transgenic MSC lines is limited. Therefore, the feasibility of genetically engineering hFOB lines was approached by applying two different methods for gene transfer, lipofection and viral-based gene transfer. hFOB were plated at low density to achieve confluency of 70–90%, and after 24 h, transfection was carried out using Lipofectamine 2000. eGFP expression was determined 48 h after transfection by means of flow cytometry. Transfection efficiency was found low, especially at elevated cell concentration (data not shown). Therefore, a hFOB cell line expressing eGFP (hFOB-eGFP) was generated with the aid of recombinant lentivirus particles delivering eGFP gene under the control of the human Uquitinase (hUbC) promoter (Fig. 2A). Stability of hFOB-eGFP was evaluated by means of flow cytometry revealing that GFP expression levels were stable over many passages (Fig. 2B). Another reporter line hFOB-hOC eGFP was generated, which enables the non-invasive monitoring of osteogenic commitment as in this line eGFP is expressed under the control of the human osteocalcin promoter (Fig. 2C). Exposure to the osteoinductive temperature of 39 °C for 3 days significantly increased eGFP expression when compared to control cultures at 34 °C (Fig. 2D).

### Mesensphere culture of hFOB

3D cell culture of MSC is preferred over 2D cell culture, since it allows cells to arrange in and self-organize an organotypic microenvironment, potentially mimicking in vivo cell-to-cell interactions and ECM organization. Cell spheroids are a model for native tissues and have the potential to support relevant physiological questions in an in vitro context. For this reason, we generated hFOB mesenspheres using both ultra-low attachment plate and in static suspension on non-adhesive plates, and culturing them for a prolonged period.

**Figure 3:**
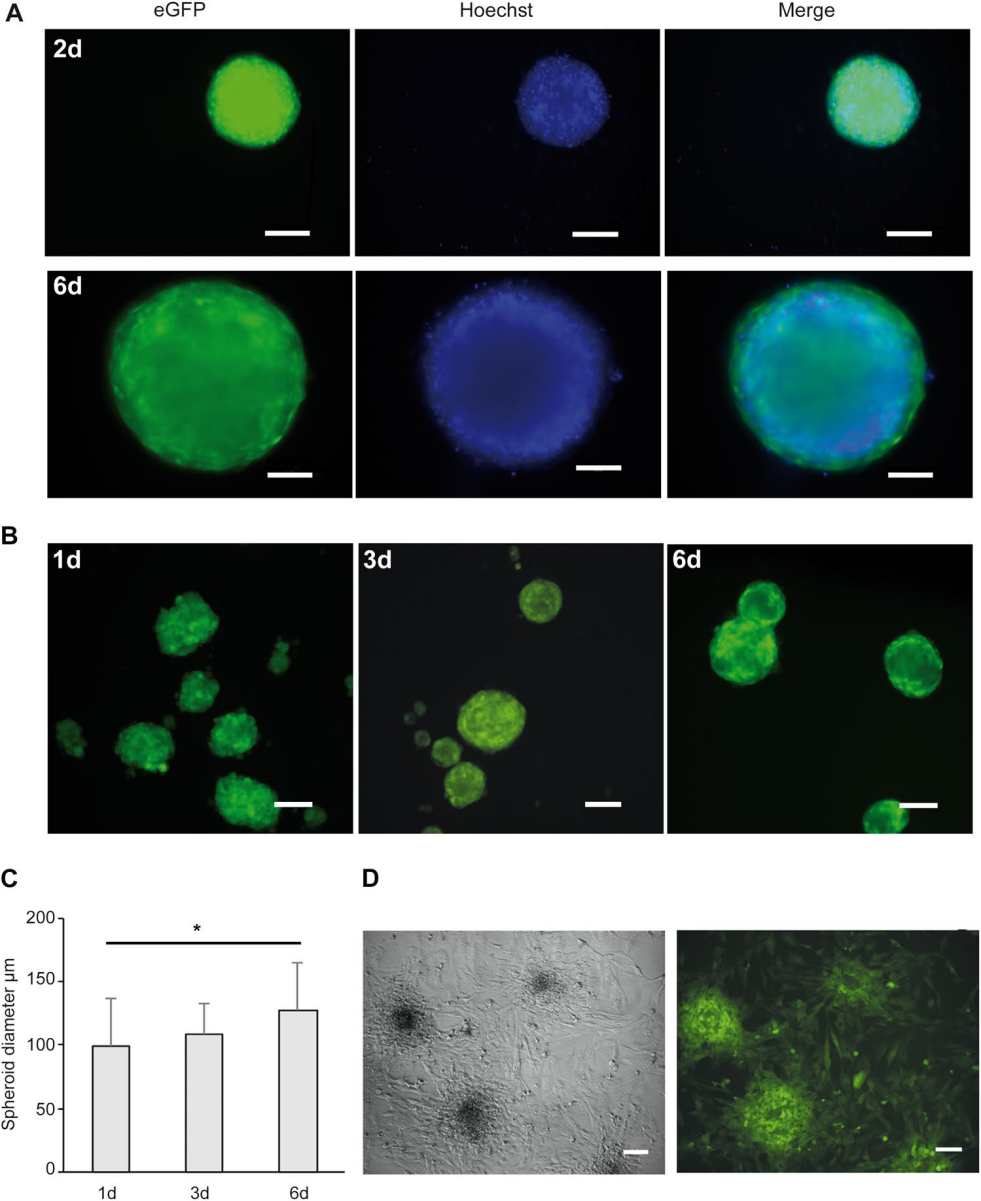
Scaffold-free spheroids. (**A**) hFOB expressing eGFP (hFOB-eGFP) were allowed to settle in low attachment 96-well U-bottom plates. After aggregation, spheroids were grown for 6 days. Living-cell staining with Hoechst was performed together with image acquisition at day 2 and 6 post-seeding. (**B**) hFOB-eGFP cells, at the density of 10^6^ cells/well, were allowed to form spheroids in static suspension in a low attachment Petri dish in the absence of serum. Serum was replaced by 15% Knockout Serum Replacement (KSR) during the entire experiment. Representative images of spheroids growing for 6 days at 34 °C 20% O_2_ (DMiL Leica). Scale bars indicate 100 µm. (**C**) The diameters of the spheroids were assessed using LAS X software (Leica). *P = 0.04. (**D**) Cell outgrowth from hFOB-eGFP spheroids obtained in KSR medium was examined one day after spheroid formation. Aggregates were transferred to complete growth medium at 34 °C 20% O_2_ in a 24-well cell culture grade plastic plate. Outgrowth of cells from spheroids was observed 3 days after transfer (DmiL, Leica, LAS X software).

3D culture was successfully established in normal growth medium as well as in knock-out medium. Serum-free medium used for culture of spheroid-MSC, maintained proliferation and multipotency comparable to serum-containing medium for adherent cultures^20,21^. In both cases, spheroids formed within 48 h (Fig. 3A,B) and their average diameter increased over time indicating active growth (Fig. 3C). The eGFP fluorescence signal faded in the center of the spheroids with time, indicating a possible reduction of vitality of the innermost parts of the cellular aggregates, which could be corroborated by histological analysis (Fig. S3).

To assess whether the generation of mesenspheres induced changes in adhesion and migration, hFOB spheroids were placed into plastic dishes, which provided a surface for cellular attachment in growth medium containing 10% FBS. Spheroids readily adhered and cells spread-out displaying a typical hFOB phenotype, i.e. single-cell migration. Interestingly, cells which were closer to the inner core did not migrate and remained in situ (Fig. 3D).

**Figure 4:**
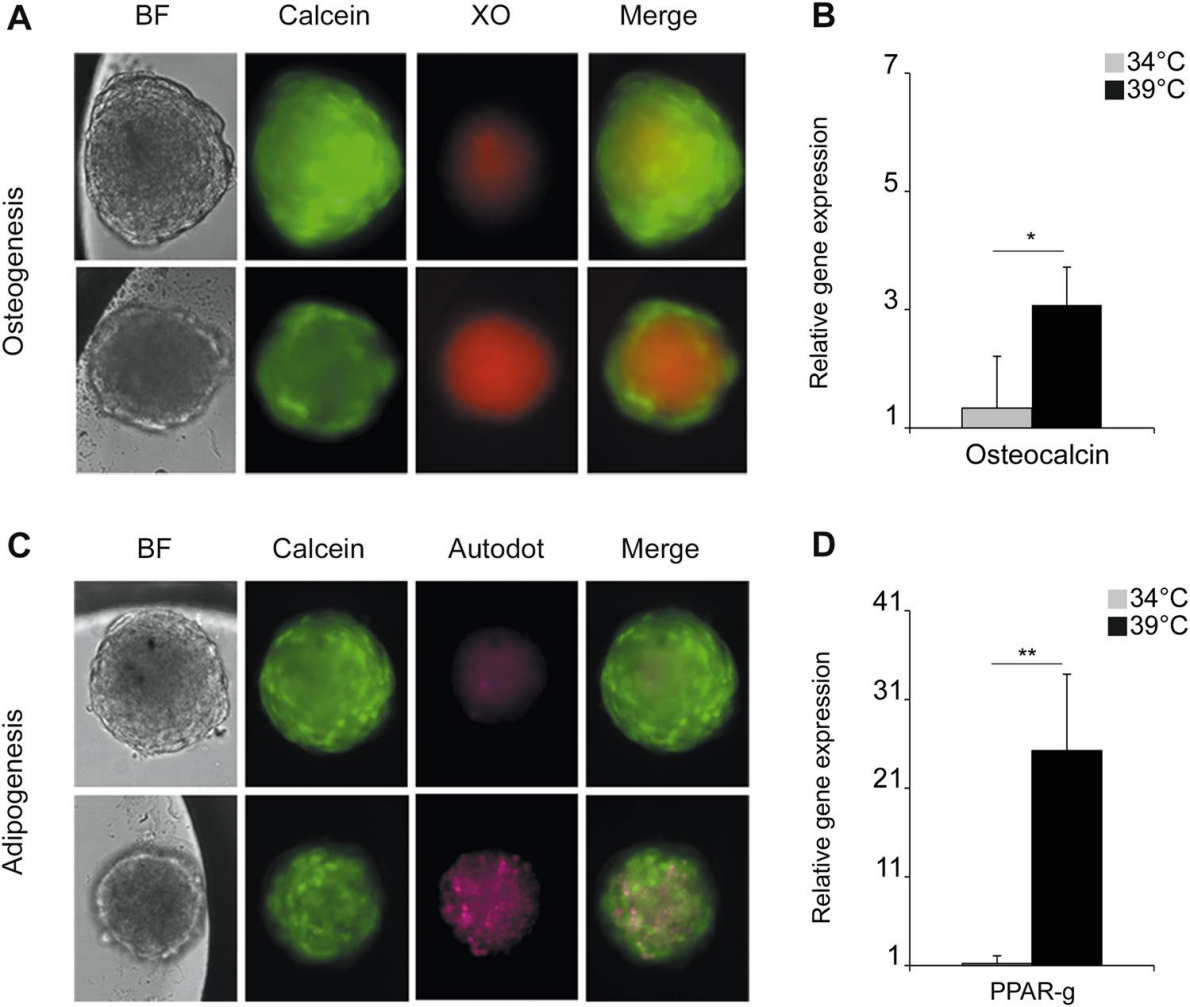
Differentiation of hFOB spheroids. (**A**) Spheroids were obtained using low attachment plates. 1000 cells were seeded per well and allowed to aggregate at the bottom of the plates for 48 h at 34 °C. Thereafter, plates were incubated for 3 days at 34 °C (upper panel) or at 39 °C (lower panel). Spheroids were stained with Calcein-AM (Calcein) and calcium deposition was visualized with Xylenol Orange (XO). For adipogenic differentiation (**C**) spheroids were incubated in adipogenic medium at 34 °C (upper panel) or 39 °C (lower panel) for 3 days. Cells were live-stained with Calcein and Autodot (pink). Images were taken using a DMi8 microscope (Leica) and acquisition analysis was performed with LAS X Software. (B, D) Quantitative PCR for the genes osteocalcin and PPARγ was performed to confirm osteogenic or adipogenic differentiation at day 4 post induction. Relative expression was normalized to hFOB cells grown in 2D at 34 °C.

Differentiation potentials of hFOB mesenspheres were also assessed. When incubated at 34 °C, they showed a larger size and exhibited a brighter Calcein signal, thus inferring higher viability and proliferation (Fig. 4A–C). As already described for 2D cultures at 39 °C, also 3D-cultivated cells ceased proliferation and commenced osteogenic differentiation as indicated by positive calcium staining and up-regulation of osteogenic marker gene expression (Fig. 4A–C; Fig. S4). Likewise, by incubation of spheroids at 39 °C in adipogenic medium, high lipid droplet content was apparent as indicated by a stronger staining with Autodot in comparison to the controls maintained at 34 °C. These findings were also supported by a robust up-regulation of PPARγ (Fig. 4B–D).

### Cell growth at variant culture conditions

**Figure 5:**
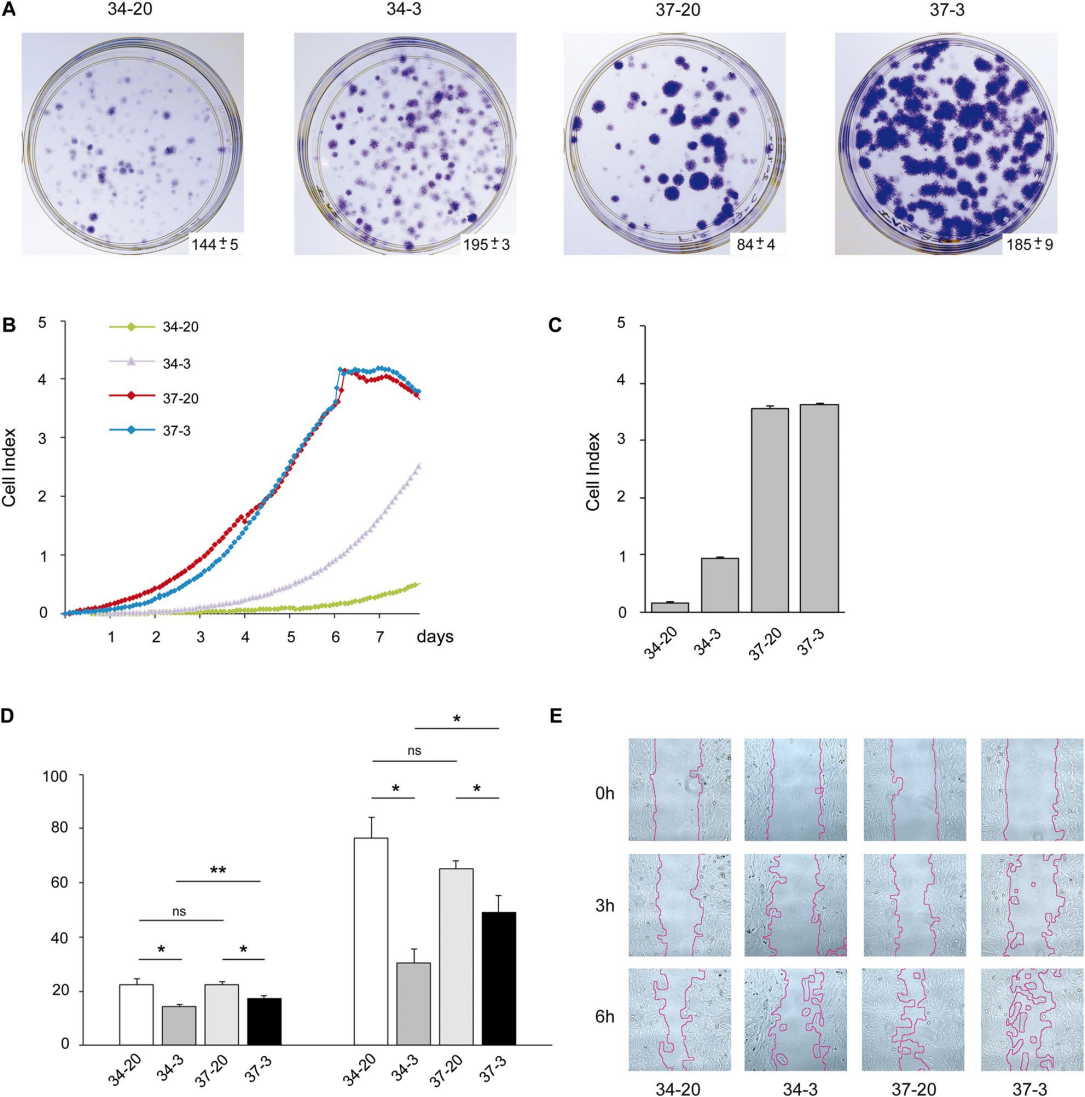
Effects of temperature and oxygen tension on hFOB properties. (**A**) hFOB cells were seeded at a density of 10 cells/cm^2^ and allowed to form colonies for 15 days at 34 °C/20% O_2_ (34–20); 34 °C/3% O_2_ (34–3); 37 °C/20% O_2_ (37–20) and 37 °C/3% O_2_ (37–3). After fixation and staining with crystal violet, colonies were counted and depicted as mean ± standard deviation (n = 3). **B)** hFOBs were seeded at a density of 2 × 10^2^/cm^2^ in each well of an impedance reader E-plate and cell growth was non-invasively monitored every 2 h over a time lapse of 8 days. Impedance was expressed as a Cell Index (CI) value. Representative graph from xCELLigence system are shown for growth curves of hFOB cultured at specified temperature and oxygen conditions. (**C**) CI-values were compared at 6 days after seeding. (**D**) Cell wounding and assessment of migratory behavior of hFOB at passages 5–7 at various culture conditions. Quantitative image data acquisition was performed with Live Cell Imager CELL IQ and analysis was performed with CellActivision Software (Yokogama). Results for quantitative analysis of percentage of gap closure at the different culture conditions are shown for 3 h and 6 h after gap opening. (**E**) Representative images at given conditions are shown. Gap closures are highlighted at 0 h, 3 h and 6 h after gap opening with pink lines.

In addition to the influence of temperature, also effects due to partial oxygen tension were monitored. Overall, the semi-permissive temperature of 37 °C supported a faster growth but negatively affected colony formation in comparison to the recommended culture temperature of 34 °C. The latter indicated an effect on potential stemness characteristics of hFOB. Therefore, hFOB monolayer grown under 20% O_2_ (34 °C), were re-seeded at a density of 10 cells/cm^2^, and were now allowed to form colonies either under 20% O_2_ (normoxic) or 3% O_2_ (hypoxic) conditions at 34 °C and 37 °C. A higher number of colonies were formed at 34 °C in comparison to 37 °C. At 3% O_2_ colony formation was favored at both temperature conditions (Fig. 5A). Colonies grown at 37 °C exhibited bigger sizes and higher densities, which indicated that the physiological temperature of 37 °C reinforced hFOB proliferation. Real-time monitoring of proliferation at 34 °C and 37 °C under normoxic or hypoxic conditions was performed using electrical impedance as the readout of an xCELLigence system. Cell growth, as an extrapolation of the Cell Index, was faster when cells were cultured at 37 °C in comparison to the permissive temperature of 34 °C. At 37 °C, proliferation rate was not affected by oxygen tension at later times (see 5–7 days post seeding) compared to 34 °C (Fig. 5B). Indeed at 34 °C, 3% O_2_ had a positive influence on growth at day 6 of culture in comparison to 20% O_2_ (Fig. 5C)_._

The semi-permissive temperature of 37 °C supported a faster growth but negatively affected colony formation when grown in low density cultures in comparison to the recommended culture temperature of 34 °C. It is however also conceivable that the temperature of 37 °C altered the conformation of the mutant tsA58 SV40 Large T antigen in a great number of cells allowing them to follow their specified differentiation along the osteogenic lineage. Nevertheless, some cells were still supported by SV40 activity and formed colonies of larger size and density due to a higher growth rate at this temperature. At 37 °C, 20% oxygen tension resulted in the lowest number of colonies while a hypoxic condition restored stemness independent of SV40 Large T antigen activity.

Also the migration potentials of cells grown either under standard oxygen tension or hypoxic condition at 34 °C versus 37 °C were investigated. Compared to 3% oxygen tension, 20% partial oxygen pressure resulted in faster gap closure irrespective of culture temperature. (Fig. 5D,E). After 6 h at 3% oxygen gap closure was more prominent at 34 °C than at 37 °C.

### Long-term culture restricts proliferation of hFOB

**Figure 6:**
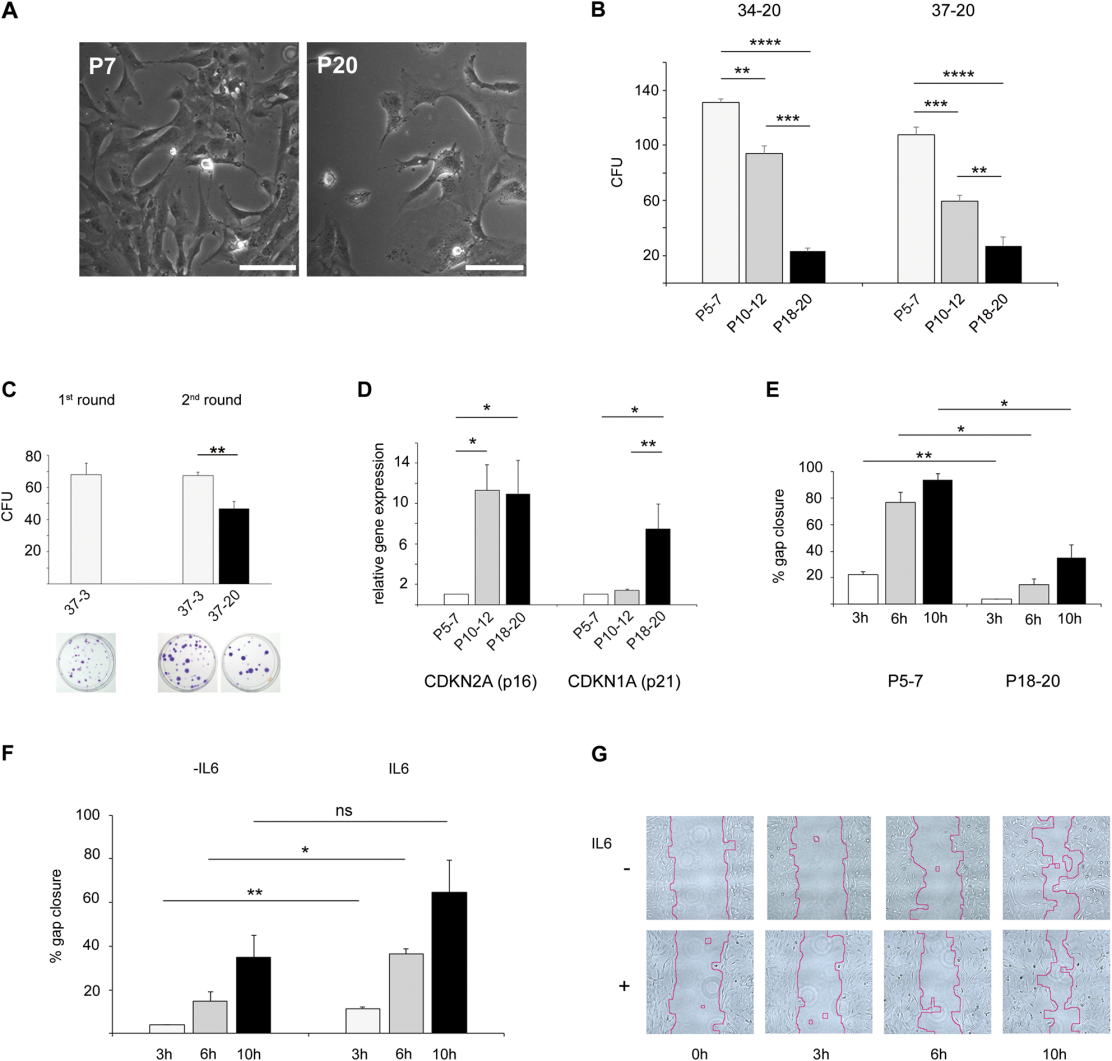
Effects of long-term culture on hFOB stemness and migratory behavior. (**A**) Brightfield microscopy (magnification × 200, scale bar 100 µm) of hFOB cells passage 7 and 20. (**B**) CFU-F of hFOB cells of passages 5–7, 10–12 and 18–20 were performed in triplicates at the given environmental settings. Cells were seeded at a density of 10 cells/cm^2^ (600 cells/plate) and incubated for 15 days. Cells were stained with Crystal violet. Mean number ± standard deviation (SD) of resulting colony-forming units fibroblasts (CFU-F) was determined. (**C**) Colony formation (2nd round) using cells derived from a previously formed colony (1st round) was performed in triplicate at the given temperature and oxygen tension. Cells were seeded at a density of 10 cells/cm^2^ (600 cells/plate) and incubated for 15 days. After the 1st round, cells were trypsinized and seeded again for a 2nd term of colony formation (density of 10 cells/cm^2^). (**D**) mRNA levels within hFOB cells at passage 10–12 or passage 18–20 encoding CDKN2A (p16) and CDKN1A (p21) were measured by RT-qPCR, normalized to YWHAZ and plotted as fold induction relative to the levels of hFOB P5-7. (**E**) Migratory behavior of passage 5–7 and passage 18–20 cells at 34 °C 20% O_2_. Determination of %-gap closure after 3 h, 6 h and 10 h. (**F**) Migration of hFOB passage 18–20 in the presence of IL-6 (10 ng/ml) and without IL-6 (CTR). Determination of %-gap closure after 3 h, 6 h and 10 h. (**G**) Representative images of the gap closure quantified in (**F**) after 0, 3, 6 and 10 h post IL-6 treatment.

Age-related changes in bmMSC include loss of stemness and proliferative capacity. These features have been also observed during long-term culture, showing a clear decline in replicative lifespan^13,22^. We therefore investigated cellular fitness in long-term culture. We noticed that at higher passages, cells underwent morphological changes and their growth rates declined. Cells at high passages (P20) showed an enlarged and more pronounced flat phenotype in comparison to those at low passage (P7) (Fig. 6A). Colony formation as an indicator for stemness was assessed at 34 °C and 37 °C in the presence of 20% oxygen. Colony forming unit (CFU) frequency was gradually reduced during subculture from low (5–7), to intermediate (10–12), to high passage (18–20), especially at 37 °C (Fig. 6B). Intermediate-passage cells from colonies generated at 37 °C and 3% O_2_ were used for a second round of colony-formation assay. Under these conditions, 3% oxygen supported a similar number of colonies as in the first round, while at 20% O_2_ secondary colony numbers were found to be significantly reduced (Fig. 6C). At higher passages, cells increased in size and proliferation decreased. Less self-renewal potential at elevated cell passage (P19 vs P6) was accompanied by (i) enhanced transcription of the cell cycle regulators p16 (CDKN2A) and p21 (CDKN1A) (Fig. 6D) and (ii) significant loss of migratory properties at 34 °C and 20% oxygen (Fig. 6E). Low-passage cells (P7) were able to significantly reduce the gap (approx. 80% closure), while high passage cells (P18) closed the gap only up to 20% (Fig. 6E).

In case of MSC it is known that pro-inflammatory cytokines such as interleukin 6 (IL6), interferon gamma (IFNγ) or Tumor Necrosis Factor alpha (TNFα) lead to increased cell migration^23^. Hence, migratory behavior of hFOB treated with 10 ng/ml of human IL6 was monitored. In comparison to mock-treated cells, IL6 significantly rescued the migration deficit of senescent hFOB at the wounded site. After 6 h at 34 °C and 20% oxygen, the gap width in IL6-exposed cell cultures was significantly smaller than the one in untreated cells (Fig. 6F,G).

## Discussion

The hFOB 1.19 cell line has been generated by introducing a temperature-sensitive isotype of the SV40 large T antigen into primary human cells isolated from fetal bone. Upon induction, hFOB cells readily undergo osteogenic differentiation, strongly activate expression of osteogenic factors and reliably form mineralized nodules^24^. Further characterization of this cell line has shown only minor chromosome abnormalities, when compared to transformed osteosarcoma cell lines. Most important however, hFOB form bone in vivo^25^. Yen and colleagues have also shown that hFOB display a specific set of cell surface markers, similar to that found in bmMSC. In addition to their predetermined osteogenic fate, hFOB are multipotent and differentiate into many other mesodermal lineages. Notably, they can also acquire a neural-like phenotype^17^. hFOB bear characteristics of skeletal stem cells (SSC) and exhibit mesenchymal multi-lineage differentiation potential, show high migratory behavior and express a large variety of cell surface antigens common to mesenchymal stromal cells. Based on these findings, hFOB appear predestined to act as a suitable cell model in human mesenchymal cell biology. A surrogate system for MSC should not only provide a reliable source of material and display a similar differentiation potential but it should encompass the behavior of primary cells as a mirror of the in vivo niche. We therefore extended these studies by further characterizing of hFOB according to attributes, which are amenable for comparative analyses providing a better understanding of MSC biology, in particular also tackling mechanisms and pathways driving MSC aging.

BmMSC are progenitor cells with high proliferation potential at an undifferentiated state. In parallel, they bear the ability to bring forth more specialized precursors, which are capable of differentiating into lineages of mesenchymal character, such as bone, fat, and various other collagenous connective tissues^26^. The recent identification of human SSC, which generate progenitor cells of bone, cartilage, and stroma, but not fat has questioned the rank of bmMSC being the stromal stem cell^27,28^. bmMSC are now considered a more committed progenitor population descending from SSC, or, they could now also be conceived a heterogeneous population comprising more restricted progenitor cell types^29^. As such there are reasons to believe that an important function of bmMSC is to establish the bone marrow niche as cell explants from adult bone marrow contain significant numbers of progenitors that are committed to the osteogenic lineage. Indeed it could be shown that upon re-implantation of selected specialized osteo-progenitors compact bone is formed but not bone marrow^29,30^. In line with that, bmMSC also bring forth pre-adipocyte-like cells.

Notably, osteogenic cell populations, which contain periostal cells or trabecular bone cells exhibit a high expression of the surface antigen, CD146 (melanoma cell adhesion molecule)^30^. CD146 is a key marker for MSC; it is generally detected in MSC isolated from various tissues and endows cells with clonogenicity and multipotency^31^. Along with CD146, CD90, also CD44, a marker for MSC stemness^32^ is highly expressed by hFOB (S1). No significant variations were observed on the expression of these three surface molecules upon long-term subculture, which is in contrast to reports on MSC (data not shown). Nevertheless, it has already been reported that immortalization via SV40 can maintain differential expression of functional markers that are present in the primary cells, among them CD44 as well as CD106^33^.

Due to the concomitant expression of MSC-associated key molecules CD90 and CD44, and their multi-lineage differentiation potentials, we infer that hFOB are in reality fetal stem cell-like cells rather than specialized osteoblasts. For all those features that hFOB have in common with MSC, we believe that the hFOB1.19 line can be considered a valuable model system for basic research in MSC. Most important in this context is the finding that hFOB acquire a senescence-like phenotype, which is in stark contrast to the well-established model of hTERT-MSC^34,35^. The nature of high-passage cells certainly needs further characterization. Nevertheless, our observations and analyses indicate that conditionally immortalized hFOB have the potential to preserve a MSC-like physiological state as would be expected under normal conditions. For this purpose we adjusted culture conditions and protocols.

The impact of long-term cultivation on cellular fitness is well documented in MSC: at a pre-senescent state, self-renewal and migratory properties drastically decline^18,36,37^. The migratory potential can be enhanced in old MSC by specific cytokines^38^, only one being interleukin-6 (IL6). This biological trait of MSC cannot be reproduced by standard cell lines, for example hTERT-MSC^39,40^, most likely because they efficiently escape senescence. This confirms its potential as a novel model as conditionally immortalized hFOB largely preserve a MSC-like cell physiological state comparable to normal in vivo conditions. The fact that long-term culture impacts the hFOB’s self-renewal and migratory capacities suggests that hFOB exhibit a higher degree of plasticity compared to most common MSC models. Also in good concordance to bmMSC, the hFOB self-renewal potential were significantly reinforced when cultivation was undertaken at atmospheric conditions of 3% O_2_^41–43^. Hypoxia supported clonogenicity of hFOB but reduced migration in a scratch assay. Although several studies demonstrated hypoxia enhances migration of MSC^44^, other authors have shown that gap closure occurred more rapidly at normoxic conditions^45,46^, comparable to our findings with hFOB.

In addition to that, the possibility to develop hFOB reporter cell lines makes them superior to MSC to study stem cell-intrinsic and extrinsic questions. This is an important feature of hFOB, because it would overcome the limitation of cell line generation in the MSC field, as MSC, similar to most primary cells isolated from adult tissues, have a finite capacity to replicate in vitro. Together with the propensity of being capable in vitro to form 3D mesenspheres, which resemble tissues formed in vivo, hFOB1.19 represent a novel means to reproduce cellular functionalities of primary MSC in the context of their microenviroment.

In conclusion, hFOB are not only resembling MSC^44,45^, but exhibit many common features, for instance when being cultivated at physiological oxygen conditions, stemness is enhanced, proliferation is stimulated and migratory behavior becomes biased. Based on our observations, it became clear that long-term cultivated hFOB gain cellular history, they refrain from immaculate self-renewal, thus they display changes, which resemble a cellular aging phenotype.

## Methods

### Cell culture

The immortalized human fetal osteoblast cell line hFOB1.19 (hFOB) was purchased from American Tissue Type Culture (ATTC) and cultured according to the protocol specified by the repository (ATCC^®^ CRL-11372™). For in vitro proliferation, hFOB1.19 cells were maintained in DMEM:Ham’s F12 (Sigma, D6421) medium supplemented with 10% fetal bovine serum (FBS), 2.5 mM l-glutamine and 300 µg/ml G418 (Sigma, G8168), designated herein growth medium. hFOB cells were cultured in a humidified incubator at atmospheric conditions of either 20% or 3% O_2,_ always supplemented with 5% CO_2_ at temperatures of 34, 37 or 39 °C.

### Immunophenotyping

For surface antigen detection, 5 × 10^5^ ells were washed and blocked for 1 h with PBS containing 4% FBS after detachment with 0.25% trypsin/EDTA. Cells were characterized with fluorescein isothiocyanate- or phycoerythrin-conjugated primary antibodies for human surface antigens and compared with appropriate isotype controls. The following conjugated monoclonal antibodies were used for hFOB immunophenotyping: CD90 PE (Biologend 5E10/328110), CD73 PE (Biolegend AD2/344003), CD45 PE (Biolegend HI30/304008), CD19 PE (Biolegend HIB19/302207), mouse IgG1k Isotype PE (Biolegend MOPC-21/400112), CD 44 PE (BD Bioscience 550989), CD106 PE (BD Biosciences 555647), CD34 PE (BD Bioscience 555822) and CD105 FITC (Ancell 326040), CD146 PE (Biolegend P1H12/361006). Flow cytometry analysis was performed using CytoFlex S with CytoExpert and Kaluza softwares (Beckman Coulter).

### Reporter cell lines

A monoclonal cell line expressing enhanced green fluorescent protein (eGFP) under the control of the human ubiquitinase (UbC) Promoter (hFOB-eGFP) was generated using the lentivirus vector FG 12^47^ (Addgene, Plasmid #14884). A transgenic reporter cell line expressing eGFP under the control of the human osteocalcin (hOC) promoter was obtained by cloning the respective sequence from hOC-pGL3 plasmid^48^ into the vector peGFP-N1 (Clontech) using KpnI and BglII restriction sites (phOC-eGFP). The hOC-eGFP cassette was excised by XhoI and AgeI endonucleases and ligated in to the lentivirus expression plasmid FG12 (Addgene, Plasmid #14884), which was digested with the restriction enzymes SalI and AgeI (pLV-hOC-eGFP). For production of recombinant lentivirus, adherent HEK 293FT cells were used for virus packaging and production. One day prior transfection, 5 × 10^6^ HEK 293 FT cells were grown in DMEM-medium containing 10% FBS (without antibiotics) in a 100 mm dish. Next, HEK293T cells in 10 ml of basal DMEM (Sigma) were transfected with 4.4 μg of expression plasmid (FG12 or pLV-hOC-eGFP), 11 μg of psPAX2 and 3.6 μg of pMD2.G plasmid using 54 μl of lipofectamine 2000 (Life Technologies) as described previously^49^. After 6 h incubation, the medium containing the transfection mixture, was replaced with 6 ml of fresh complete DMEM (10% FBS, 1% l-glutamine) without antibiotics and the cells were further incubated for another 48 h at 37 °C with 5% CO_2_ in a humidified atmosphere. The virus particles were harvested by collecting the supernatant after centrifugation at 2000×*g* for 10 min at 4 °C. The supernatant was filtered through a 0.45-μm syringe filter and stored at − 80 °C.

Reporter cell lines were generated by transducing hFOB with the corresponding viral vector at a multiplicity of infection (MOI) of 5–10. Transduction was performed by centrifugation at 1000×*g* for 50 min at RT and transduced cells were expanded and monitored for eGFP expression. Reporter cell lines were sorted with FACS Aria III (BD Biosciences). hFOB-hOC eGFP cells were sorted according to a low eGFP expression and amplified as a polyclonal cell line.

Reporter activity of hFOB-hOC eGFP was assessed by comparing eGFP expression of cells incubated at the non-permissive temperature of 39 °C with their basal activity at 34 °C. Briefly, cells were seeded into 24-well tissue culture plates at a density of 10^5^ cells/well. After incubation overnight at 34 °C to allow attachment, cells were transferred to the non-permissive temperature of 39 °C for 3 days. Control cells were left at 34 °C. Emissions of green fluorescence were visualized by fluorescence microscopy (DMIL, Leica) and quantified by flow cytometry using Cytoflex S and Kaluza Analysis software (Beckman Coulter).

### 3D culture

Mesensphere were generated in ultra-low attachment U-bottom 96-well plate (Greiner). Plates were coated with sterile-filtered 3% pluronic F-127 (Sigma, P2243) in PBS and were either used the same day or aseptically stored at 4 °C. Cells were seeded at the indicated concentration (10^3^–10^4^ cells/well) and allowed to aggregate into spheroids for 48 h in growth medium at 34 °C and 5% CO_2_/20% O_2_ before further processing. For induction of differentiation, after the sedimentation phase, spheroids were transferred to 39 °C and 5% CO_2_/20% O_2_ either in growth medium (osteogenesis), or in adipogenic medium as indicated in the section “Differentiation” studies. For experiments in serum-free medium, fetal bovine serum was substituted in the growth medium with 15% KnockOut™ Serum Replacement (KSR) (Gibco, #10828028). 10 ml of a cell suspension containing 5 × 10^5^ cells/ml in KSR-medium were added to a sterile uncoated culture dish exhibiting a surface repellent to cells, thereby forcing cells to form spheroids, which in due course can be also grown under this conditions.

### Immunofluorescence, cytochemical staining and histology

Accumulation of extra-cellular calcium was assayed using Xylenol Orange (Fluka, #33825) as a living-cell dye, or alternatively after fixation with Alizarin Red (Fluka, #440563). Xylenol Orange was dissolved in water to a 5 mM solution, and sterile-filtered using a 0.22-µm filter. Samples were incubated overnight at a final concentration of 20 µM. Before imaging, cells were washed and growth medium was added to prevent non-specific background fluorescence. Mineral content was also detected by Alizarin Red staining. Cells were washed and fixed with 4% paraformaldehyde for 30 min. Alizarin Red S staining (2% aqueous solution pH to 4.1–4.3) was performed for 45–60 min at room temperature.

Autodot (Abcepta, #SM1000a) was applied to label lipid droplet formation as described previously^50^. Briefly, spheroids were first washed with PBS and incubated with 100 µM Autodot for 1 h. After three washes with PBS, spheroids were transferred into phenol-red free medium for imaging.

### Quantitative PCR

Total RNA was isolated with the RNeasy Plus Mini Kit (Qiagen), quantified with a NanoDrop 1000 spectrophotometer (Thermo Scientific) and stored at − 80 °C. Complementary DNA (cDNA) synthesis was performed with 100–500 ng of total RNA using the RevertAid First Strand cDNA Synthesis Kit (Thermo Scientific). Quantitative PCR was performed on an AriaMX Real-Time PCR System (Agilent) with the Luna Universal qPCR Master Mix (New England Biolabs). All PCR reactions were prepared three times in duplicates. To determine primer specificity, melting curves were performed after 40 cycles of PCR. Fold difference in gene expression was calculated using the ΔΔCt method and normalization according to the expression of the references gene *YWHAZ*. Primer sequences are given as supplement (SI, Table).

### Differentiation

All differentiation experiments were conducted at 39 °C and 5% CO_2_/20% O_2_. For osteogenic induction, monolayers were incubated at 34 °C and 5% CO_2_/20% O_2_ in growth medium until the cell lawn reached confluency. The culture was then transferred to 39 °C and 5% CO_2_/20% O_2_. In the case of adipogenic differentiation, incubation was performed in adipogenic medium consisting of growth medium with the addition of 0.5 mM isobutyl‐methylxanthine (Sigma, I5879), 1 μM dexamethasone (Sigma, D4902), 10 μM insulin (Sigma, l9278), and 60 μM indomethacin (Sigma, I7378).

### Colony formation

In order to assess colony formation, 10 cells were seeded per cm^2^ in triplicates in a 100 mm culture plate. The colonies were counted after 15 days in culture and the average number of colonies of three plates was calculated. A colony was defined as consisting of at least 50 cells. The analysis was performed for different culture conditions or cell passages.

An iterated colony forming assay was performed as follows: colonies obtained at 37 °C 3% O_2_ from an intermediate cell passage (p12) were detached and subjected to a second term of colony formation. 10 cells/cm^2^ were seeded and plates were incubated either at 37 °C/3% O_2_ or 37 °C/20% O_2_ for 15 days in the presence of G418.

### Real-time impedance-based monitoring of cell proliferation

Cell proliferation was assessed by means of the online monitoring xCELLigence system equipped with an E-Plate (ACEA Biosciences Inc.). 100 cells were seeded in each well of an E-plate in growth medium. The impedance value of each well was automatically monitored by the device every 2 h for the duration of 180 h and expressed as a cell index, or CI-value. Impedance was acquired for four different culture conditions: 34 °C at 20% O_2_, 34 °C at 3% O_2_, 37 °C at 20% O_2_, and 37 °C at 3% O_2_.

### Cell migration

hFOB migration capability was assessed as follows: a 2-well silicone insert (Culture-insert 2 well, Ibidi) was placed in a 12-well microtiter plate and used to create a defined cell-free gap of 500 ± 100 µm. hFOB cells (2 × 10^5^ cells in 70 µl growth medium) were seeded in each reservoir and incubated overnight. In case of IL6 treatment hFOB cells were pre-treated with IL6 (10 ng/ml) before seeding in the culture insert. IL6 treatment was maintained throughout the entire migration assay. After cell attachment, the insert was removed the following day and cell migration was analyzed by evaluating gap closure over 6 h using a live-cell imager (Cell IQ, Chip-Man technologies). The progress of gap closure was assessed every 30 min by obtaining photomicrographs starting at the initial time point until completion of the study.

The rates of gap closure were analyzed by applying the scratch wound measurement tool of the CellActivision software (Yokogawa Electric Corporation). Rates of gap closure were determined both by assessing gap width over time, as well as by quantifying the area devoid of cells over time. All assays were performed in triplicate.

### Statistical analysis

All experiments were carried out independently for at least three times. Data were expressed as mean ± standard deviation. Statistical significance was evaluated using a two-tailed Student’s *t* test and P values of ≤ 0.05 were considered significant (*), whereas a significance of ** refers to p ≤ 0.01 and *** denotes a p ≤ 0.001, respectively.

## Supplementary Information

Supplementary Information.
