# Supplementary material for: Kinship of conditionally immortalized cells derived from fetal bone to human bone-derived mesenchymal stroma cells
Source: Sci Rep. 2021 May 25;11:10933. doi: 10.1038/s41598-021-90161-2 (PMC8149839; doi:10.1038/s41598-021-90161-2)
Supplement: Supplementary file 1 — Supplementary Information. [file 41598_2021_90161_MOESM1_ESM.docx]

**Supplementary Information**

Kinship of conditionally immortalized cells derived from fetal bone to human bone-derived mesenchymal stroma cells

**Marozin S*.^1^, Simon-Nobbe B*.^1^, Irausek S.^1^, Chung L.W.K.^2^ and Lepperdinger G.^1^**

**Supplemental Fig.S1 gene expression of osteogenic markers in hFOB cultured at 39°C**


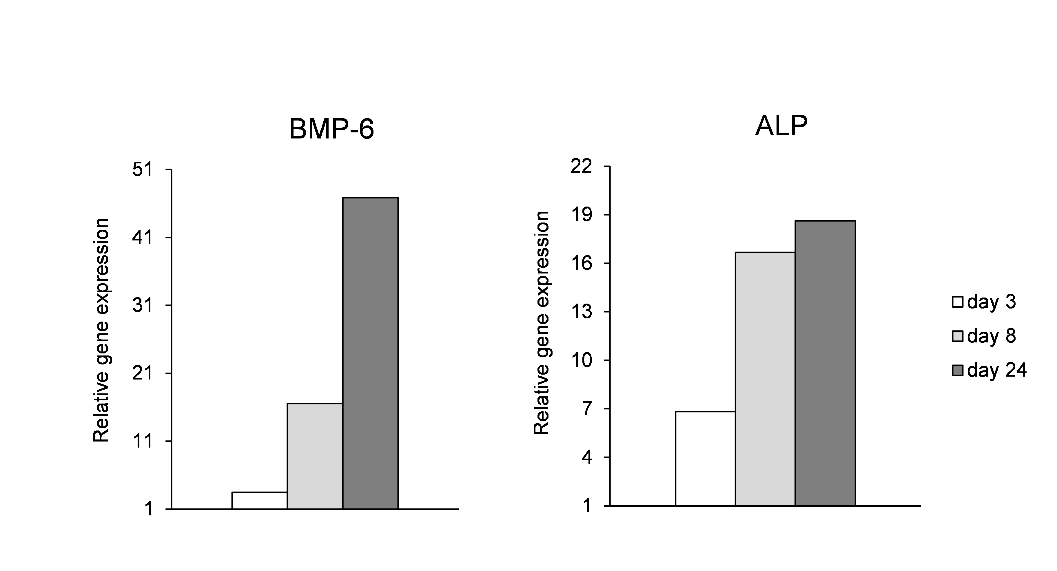


**Fig.S1 Osteogenic-marker expression in hFOB cultured at 39°C.** Relative expression of Bone Morphogenetic Protein (BMP)-6 and alkaline phosphatase (ALP). Quantification was performed by means of qRT-PCR in reference to the transcriptional levels of YWHAZ. Increased expression of these molecular markers indicates osteogenic induction in hFOB cultivated at elevated ambient temperature commencing already after three days.

**Supplemental Fig.S2 CD146 expression in hFOB and hMSC**


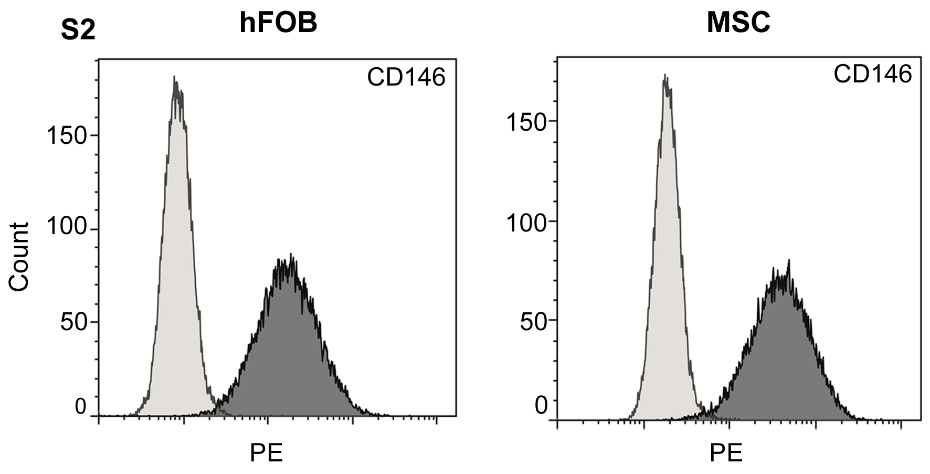


**Fig.S2 CD146 expression.** hFOB1.19 were compared to bone-derived human mesenchymal stem cells. Staining with CD146 phycoerythrin-conjugated (PE) primary antibodies (dark grey) and flow cytometric analyses showed that both cell types presented elevated levels of CD146 at their surface. Results of treatment with antibody isotype controls are shown in light grey.

**Supplemental Fig.S3 Histological analysis of hFOB spheroids**

**
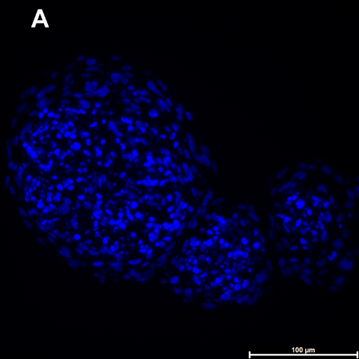

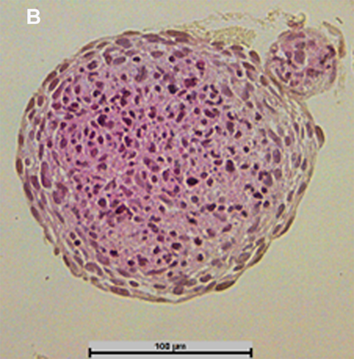
**

**Fig.S3 Histological analysis of hFOB spheroids:** Paraffin sections of 6 days-old hFOB spheroids: (**A**) Staining with DAPI (blue) highlight the cell nuclei, (**B**) hematoxylin-eosin staining contrast-enhanced cellular morphology, showing that cells compacted of the core of the aggregate are surrounded by a sheet of cells exhibiting epithelial characteristics.

**Supplemental Fig.S4 gene expression of osteogenic markers in hFOB spheroids**

**
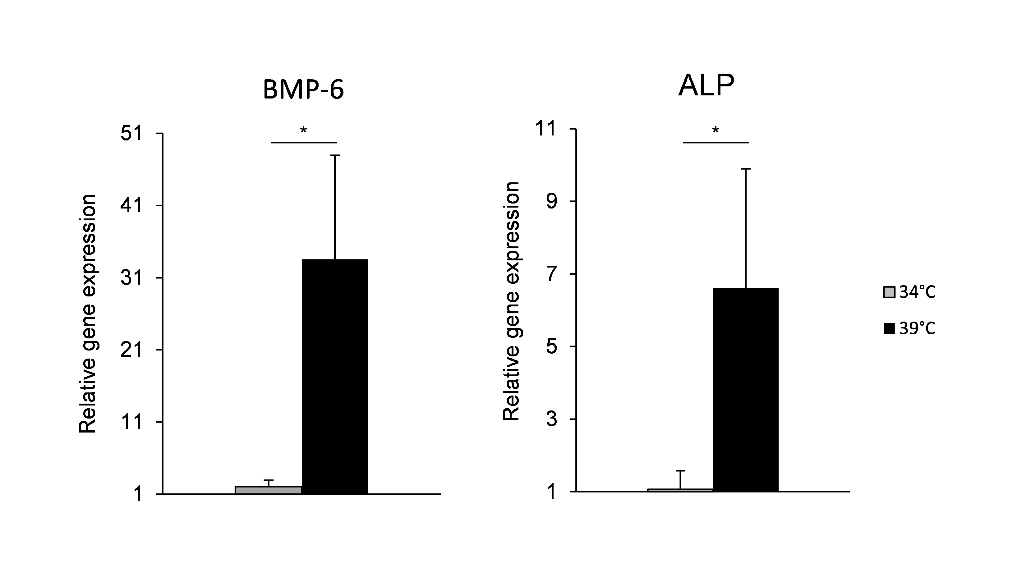
**

**Fig.S4 Differential expression of osteogenic-markers in hFOB grown as spheroids.** Relative transcriptional levels of Bone Morphogenetic Protein (BMP)-6 and alkaline phosphatase (ALP) was assessed in hFOB spheroids cultured at 34°C or 39°C after a sedimentation time of 48 hours at 34°C. Expression was normalized to transcriptional levels assessed in hFOB, which were grown under standard conditions, i.e. grown as a cell lawn in growth medium at 34°C. * p<0.05. Elevated expression of the selected molecular markers indicated induction of osteogenesis at elevated temperature.

**Table S1:** Primers applied in RT-PCR analyses.

| Gene target | Forward primer (5’ – 3’) | Reverse primer (5’ – 3’) |
| --- | --- | --- |
|  |  |  |
| Osteocalcin | GCAGCGAGGTAGTGAAGAGA | GGAGGTCAGGGCAAGG |
| ALP | CTTCAAACCGAGATACAAGC | TCAGCTCGTACTGCATGTC |
| BMP-6 | CAG CCT GCA GGA AGC ATG AG | CAA AGT AAA GAA CCG AGA TG |
| PPARγ | TACATAAAGTCCTTCCCGCTGA | GGGGTGATGTGTTTGAACTTG |
| CDKN1A-p21 | ATGTGGACCTGTCACTGTCTT | GTGGTAGAAATCTGTCATGCTG |
| CDKN2A-p16 | CCAACGCACCGAATAGTTACG | GCGCTGCCCATCATCATG |
| YWHAZ | AAGTTCTTGATCCCCAATGCTT | GTCTGATAGGATGTGTTGGTTGC |
